# Current status of the surgical training system in Japan: results of a nationwide questionnaire survey of graduating surgical trainees

**DOI:** 10.1007/s00595-024-02884-0

**Published:** 2024-06-26

**Authors:** Yoshiyuki Kiyasu, Saseem Poudel, Daisuke Koike, Jun Watanabe, Ryosuke Kowatari, Masayuki Fukumoto, Yoshiko Yamaoka-Fujikawa, Yuta Kikuchi, Keisuke Arai, Eisuke Booka, Sonoko Ishida, Shinichiro Yokoyama, Mitsue Saito

**Affiliations:** 1 Department of Surgery, Kyoto University Graduate School of Medicine, 54 Shogoin-Kawahara-cho, Sakyo-ku, Kyoto, 606-8507 Japan; 2 Department of Gastroenterological Surgery II, Hokkaido University Graduate School of Medicine, Kita 15 Nishi 7, Kita-ku, Sapporo, Japan; 3 Department of Gastroenterological Surgery, Fujita Health University School of Medicine, BANTANE Hospital, Aichi, Japan; 4 Department of Surgery, Division of Gastroenterological, General and Transplant Surgery, Jichi Medical University, Shimotsuke, Tochigi Japan; 5 Department of Thoracic and Cardiovascular Surgery, Hirosaki University School of Medicine, Aomori, Japan; 6 Department of Surgery, Nagasaki University Graduate School of Biomedical Sciences, Nagasaki, Japan; 7 Department of Enterology, Fukamidai Medical Clinic, Kanagawa, Japan; 8 Department of Surgery, Otakanomori Hospital, Chiba, Japan; 9 Department of Surgery, Division of Hepato-Biliary-Pancreatic Surgery, Kobe University Graduate School of Medicine, Kobe, Hyogo Japan; 10 Department of Surgery, Hamamatsu University School of Medicine, Shizuoka, Japan; 11 Department of Surgery, Kita-Harima Medical Center, Hyogo, Japan; 12 Department of Pediatric Surgery, Hokkaido Medical Center for Child Health and Rehabilitation, Sapporo, Japan; 13 Department of Breast Oncology, Juntendo University Graduate School of Medicine, 2-1-1 Hongo, Bunkyo-ku, Tokyo, 113-8421 Japan

**Keywords:** Work–life balance, Quality of life, Nationwide survey, Surgical education, Surgical residency training

## Abstract

**Purpose:**

To identify the problems trainees face during surgical training in Japan.

**Methods:**

A nationwide online questionnaire survey was conducted targeting newly certified surgical trainees.

**Results:**

The response rate was 53.8% (758/1410). Among those respondents, 25.6% were women, 71.4% were either married or had a partner, 41.3% had children, 72.7% had performed over 200 surgeries under general anesthesia, and 54.1% had chosen, before graduating from medical school, to become a surgeon. While 88.8% were interested in learning surgical techniques, 63.8% were hesitant to become a surgeon for fear of a compromised quality of private life (QOL). Conversely, only 1.4% chose their surgical training programs based on QOL. Overall, 84.6% of the trainees were satisfied with their training and this correlated with the number of surgeries performed. Only 29.9% received non-technical skill training. The average number of night shifts per month was 5.6, and 10.6% worked over 80 h per week. Harassment was reported by 41.5% of the respondents. Moreover, 33.0% had considered dropping out at some time, primarily because of their QOL (51.1%) or the harassment they had encountered (50.4%).

**Conclusion:**

This survey revealed that while trainees were satisfied with the overall training system, issues such as long working hours and harassment are prevalent. Working to improve these issues could make surgery more attractive for young trainees.

**Supplementary Information:**

The online version contains supplementary material available at 10.1007/s00595-024-02884-0.

## Introduction

In Japan, to become a board-certified surgeon, medical graduates must first complete a 2-year compulsory internship program. During this internship, they need to apply for a surgical training program that meets the standards established by the Japan Surgical Society (JSS) [1]. Since 2018, the standards have been overseen by the Japan Medical Specialty Board [2]. This is the first step to becoming a cardiovascular, thoracic, gastrointestinal, breast, endocrine, or pediatric surgeon. Despite an increase in the number of interns in Japan, the number of applicants for surgical training programs decreased from 934 in 2021 to 835 in 2023 [1]. Unlike many countries where medical graduates undergo a competitive selection process for acceptance into surgical training [3], the total number of surgical training positions in Japan exceeds the number of applicants, as reflected in a low fulfillment rate of 39.1% (835/2135) in 2023 [1]. This decrease in the number of applicants suggests that the number of trainees interested in surgery is declining. In fact, a recent report showed that the number of surgeons has decreased by more than 1000 in the past 20 years [4]. Factors such as long working hours, compromised quality of private life (QOL), and a poor work–life balance (WLB) associated with the surgical profession are thought to contribute to this trend [5]. Although the Japanese work culture is notorious for being harsh, more importance is now being placed on QOL and WLB, especially by the younger generations in Japan. In 2024, the Japanese government made workstyle reform for medical doctors a priority of the Japanese labor policy. Given these social circumstances, it is necessary to incorporate the changing perspectives into an evaluation of the surgical training system.

Unlike in North America, surveys on surgical training have been rarely conducted in Japan [6]. In 2016, the JSS conducted a survey targeting newly certified surgical residents and their program directors to assess the surgical training programs [7–10]. The results revealed a correlation between the number of surgeries performed by trainees and the self-assessed competency and a shortage of off-the-job training. However, this survey did not include questions on family background, such as marital status, or on issues related to QOL, WLB, or harassment during training. While studies have been done on the QOL and WLB of practicing surgeons in terms of the gender gap in Japan [11–13], items encompassing a wide range of living and working environments of surgical trainees in Japan have rarely been studied. Consequently, the JSS Education Committee has opted to undertake a comprehensive nationwide online survey to systematically identify and address any prevailing challenges or issues within the current training programs. The purpose of this survey is to clarify the overall status of the surgical training system in Japan and to unveil prevailing issues surgical trainees experience during their training.

## Methods

### Ethical issues

This research proposal was approved by the JSS Research Ethics Review Committee (JSS2023-1). At the start of the survey, the participants were given a detailed explanation about the study. Only participants who gave their consent proceeded with the survey. To ensure the anonymity of respondents, no personal data that could identify them, such as name, email address, training hospital or IP address, were collected.

### Development of the survey

The JSS Education Committee called for applications from its members under the age of 40 to be part of the Under 40 working group (U-40). From among the applicants, 71 surgeons were selected to be part of the U-40. They were divided into 11 small groups of six or seven, according to their individual interests, such as education, WLB, uneven distribution of medical doctors, and research. Each group was assigned a topic related to the various challenges faced by surgical trainees and young surgeons, described above as members’ interests. Each group held online meetings every few weeks to develop questions to be included in the questionnaire survey. An editorial committee consisting of a representative from each group discussed the importance of each question in the pool of questions received from each group and decided which questions to select. Several group discussions were held to edit the questions. The survey draft was reviewed by all working group members and by the JSS Education Committee. The questionnaire was finalized after incorporating the suggestions from the U-40 group members and approved by the JSS Board of Directors.

### Questionnaire

The final questionnaire consisted of 43 questions inquiring about the “Experience of the surgical training” (Online Resource 1). It comprised the following:

1. Demographic background of the participants.
2. Motivation for joining the surgical training.
3. Assessment of the training program.
4. Regional disparity.
5. Working environment during the training, including questions about the number of working hours, overtime work in addition to the normal 40 h per week, harassment, and thoughts about quitting.

### Participants

The participants were trainees who completed their surgical training in 2021 and 2022. Because of the coronavirus disease 2019 pandemic, the certification test for both years was conducted in 2022, and all graduating trainees were certified together in 2023.

### Online survey

The 43 survey questions were uploaded on an online platform created using the SurveyMonkey online tool (SurveyMonkey Inc., San Mateo, CA, USA, www.surveymonkey.com). The JSS secretariat sent links to the survey via email to the email address the participants had provided when registering for the training program. The survey was open from April to May 2023 and three email reminders with a link to the online survey were sent during this period. None of the questions were mandatory to answer, and participants could skip any questions that they did not want to answer. Only the questions they answered were analyzed. Only one response could be recorded from one device. The system recorded the answers as they were entered and the respondents could cancel their answer anytime before the final submission. However, after the final submission, participants were unable to retract their answers. This was because the survey was completely anonymous and no information that could identify the participants or link them to their answers was collected.

### Statistical analysis

Descriptive statistical analysis was performed on the collected data using Microsoft Excel 2019. Missing data were excluded, but the rest of the answers were included into the data analysis. All data are presented as the number of respondents (and percentages) and continuous variables are expressed as the mean ± standard deviation (SD). General satisfaction according to the number of surgeries performed was analyzed.

## Results

### Demographics of the respondents

Emails with the survey link were sent to all 1410 surgical trainees who obtained their certification in 2023, and 758 (53.8%) responded to the survey. Among the respondents, 25.6% were women, 71.4% were either married or had a partner, and 53.8% answered that their spouse or partner was engaged in full-time employment (Table 1). Regarding surgical experience, 72.7% had performed over 200 surgeries under general anesthesia, 13.8% m had completed over 500 surgeries, and 52.8% were planning to pursue gastroenterological surgery as a subspeciality.

**Table 1:** Demographics of the surgical trainees who responded to the survey

Demographic characteristic	Number of respondents *n* (%)
Post-graduate years (years)	
6:7:others	315 (41.8): 335 (44.5): 103 (13.7)
Age (years)	
≦30	117 (15.4)
31–35	573 (75.6)
36–40	57 (7.5)
> 40	11 (1.5)
Year of application for board certification test	
2021:2022	292 (38.9): 458 (61.1)
Number of women	194 (25.6)
Marital status married/with partner	541 (71.4)
Number of respondents with child(ren)	313 (41.3)
Full-time working spouse/partner	256 (53.8)
Training area	
Urban^a^: regional city^b^: rural^c^	318 (42.0): 410 (54.2): 29 (3.8)
Home area	
Urban^a^: regional city^b^: rural^c^	311 (41.0): 365 (48.2): 82 (10.8)
Longest training institution	
Academic: tertiary: other: multiple	162 (21.4): 232 (30.6): 238 (31.4): 125 (16.5)
Number of general anesthesia operations	
< 100	28 (3.7)
101–200	178 (23.6)
201–300	225 (29.8)
301–400	164 (21.7)
401–500	56 (7.4)
501 ~	104 (13.8)
Subspecialty of interest	
Gastroenterological surgery	399 (52.8)
Cardiovascular surgery	86 (11.4)
Thoracic surgery	85 (11.2)
Breast surgery	85 (11.2)
Others	101 (13.4)

^a^Major city (1 million people or more), ^b^regional city (over 50,000 people), ^c^underpopulated area (under 50,000 people)

### Motivation for each surgical training program

**Fig. 1:**
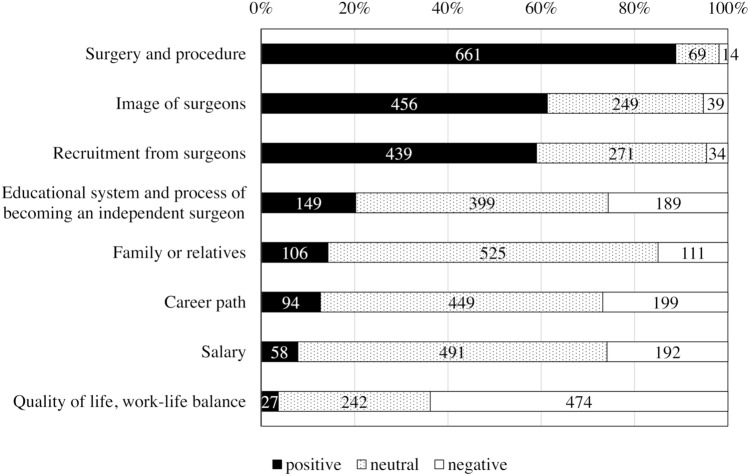
Impact of factors that influence becoming a surgeon. Each field indicates a positive, neutral, or negative impact on the choice of becoming a surgeon. The numbers in the fields indicate the number of respondents for each field

Fifty-four percent of the respondents decided to become a surgeon before graduating from medical school (Table 2). Interests in surgery and surgical procedures played a positive role in pursuing surgery for 88.8% of the respondents, whereas compromised QOL and poor WLB played a negative role for 63.8% (Fig. 1). On the other hand, the atmosphere of the workplace (22.0%), the workplace being their alma mater (21.6%), and the volume of surgical cases (17.1%) had the biggest influence on choosing their training programs, while QOL and WLB were negligible (1.4%).

**Table 2:** Applicants’ background before entering the surgical training program

Background characteristics	Number of respondents *n* (%)
When did you decide to become a surgeon?	
Before medical school	121 (16.2)
During medical school	284 (37.9)
During internship years	322 (43.0)
Other	22 (2.9)
Most influential factor for choosing a training program	
Good atmosphere	161 (22.0)
Graduating university	158 (21.6)
Volume of surgeries	125 (17.1)
Quality of life, work–life balance	10 (1.4)
Other	278 (38.0)

### Assessment of training and education

**Fig. 2:**
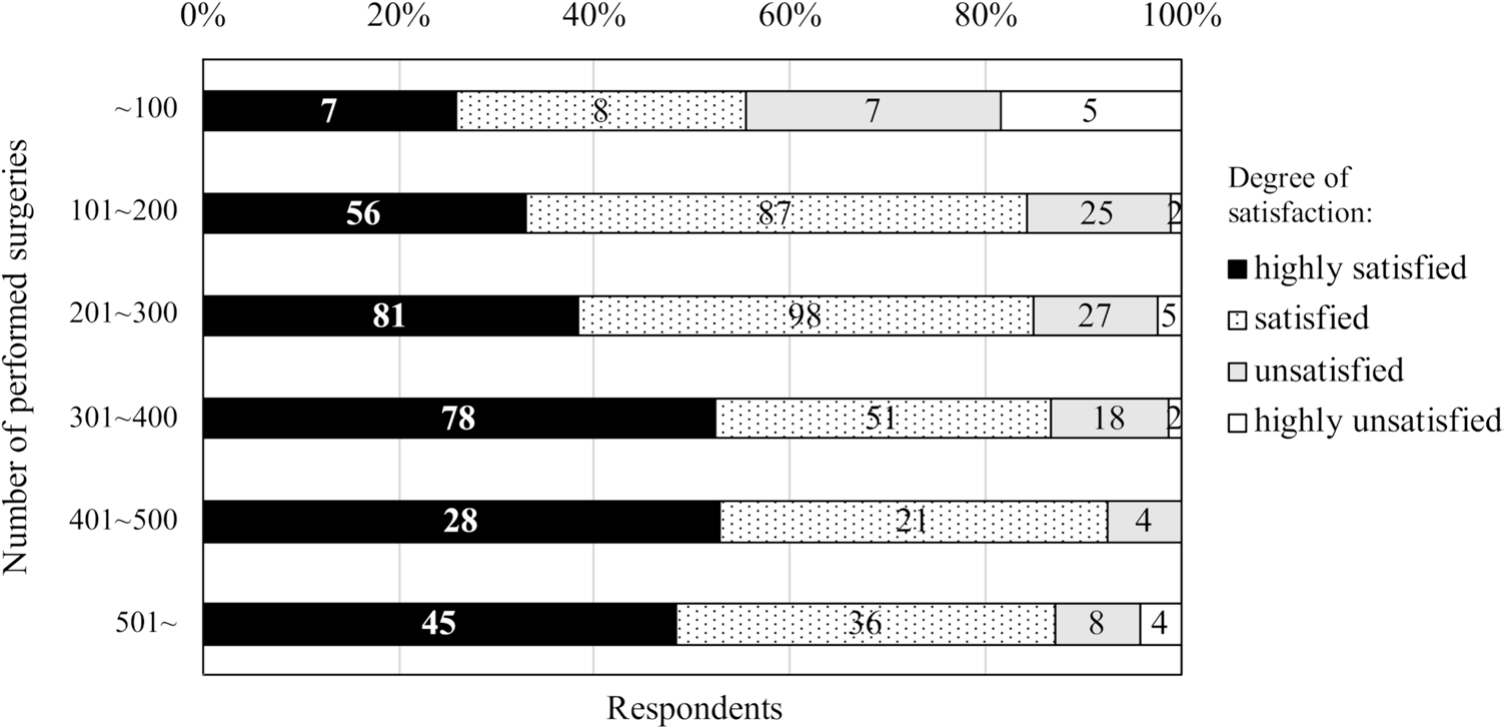
Degree of satisfaction with the training in relation to the number of surgeries performed. The numbers in the fields indicate the number of respondents for each field

Satisfaction with each surgical training program was rated as high (42.0%) and satisfied (42.6%), respectively (Table 3). The number of surgeries performed correlated with the degree of satisfaction, except for the group with the largest number of surgeries (Fig. 2). The attending surgeons’ clinical skills were rated as very high or high by 96.7%, while their educational skills were evaluated as very high or high by 84.1%. Regarding off-the-job training, 88.5% had received technical skill training, while 29.9% had received non-technical skill training (Table 4).

**Table 3:** Evaluation of training results

Item for evaluation		Number of respondents *n* (%)
Satisfied or very satisfied with their training program		621 (84.6)
High or very high evaluation of attending surgeon as a clinician		711 (96.7)
High or very high evaluation of attending surgeon as an educator		618 (84.1)
Number of published papers		
None: 1: 2–4: > 5	169 (23.1): 241 (32.9): 260 (35.4): 63 (8.6)	
Limiting elements to publishing a paper		
Practical knowledge on how to write/submit		453 (61.9)
Time allocatable to writing		346 (47.3)
Motivation or interest		317 (43.3)
Clinical knowledge		271 (37.0)
Data/cases		241 (32.9)
Mentor		221 (30.2)
Co-author(s)		131 (17.9)
Nothing was limiting		7 (1.0)

**Table 4:** Off-the-job training during the surgical training program

Frequency of training	Technical skills	Non-technical skills
Undertaken regularly	127 (17.3)	50 (6.8)
Undertaken inconsistently	523 (71.2)	169 (23.1)
Did not take the opportunity	18 (2.4)	27 (3.7)
Did not have the opportunity	61 (8.3)	311 (42.4)
Unsure	6 (0.8)	176 (24.0)

Regarding academic education, 23.1% had never published a scientific paper. A shortage of practical knowledge was reported by 61.9%, followed by a lack of research time (47.3%) and low motivation (43.3%). One-third to half of the trainees had never watched educational lectures provided by the JSS (Table 5). On the other hand, the trainees tended to utilize materials offered by third parties.

**Table 5:** Degree of usefulness of various educational materials

	Useful	Not useful	Never used
Editorial and treatment video lectures offered by the JSS^a^	53.5	7.3	39.2
Procedure lectures DVD offered by the JSS	45.3	6.5	48.2
Research video lectures offered by the JSS	35.8	9.1	55.1
Trauma video lectures offered by the JSS	52.4	11.4	36.3
Editorial and treatment video lectures offered by a third party	76.3	6.0	17.7
Procedure video lectures offered by a third party	82.0	3.5	14.5
Research video lectures offered by a third party	45.7	8.1	46.3
Japan Advanced Trauma Evaluation and Care Training Course^b^	64.3	9.5	26.1

Data are expressed as percentages (%)

^a^JSS; Japan Surgical Society

^b^Available at: https://www.jtcr-jatec.org/index_jatec.html

In response to Japan’s low incidence of trauma cases, a point-based system was implemented in 2016, allowing for the earning of points through courses and simulation education in addition to surgical cases. Despite these measures, 39.7% of respondents reported trauma as the most challenging area to meet program requirements (Table 6). Moreover, 26.2% felt that the training content did not meet the training aims, and 24.0% thought trainees did not understand the curriculum content.

**Table 6:** Problems with the training program

	Number of respondents *n* (%)
What do you think are the problems with the training program? (multiple answers allowed)	
Nothing	290 (39.8)
Mismatch of training contents and target	191 (26.2)
Curriculum content not understood by trainees	175 (24.0)
Inadequate feedback from instructors	124 (17.0)
The training program of the facility is not evaluated	95 (13.0)
Others	129 (17.7)
In which area did you find it difficult to collect the required minimum number of cases?	
Area (requirement)	
Trauma (10 points)	292 (39.7)
Pediatric surgery (10 cases)	91 (12.4)
Cardiovascular (10 cases)	90 (12.2)
Others	150 (20.4)
No difficulty	112 (15.2)
Have you ever been harassed by the attending surgeons?	
Yes	304 (41.5)
No	381 (52.0)
Prefer not to answer	48 (6.6)
Have you ever considered quitting the program during your surgical training?	
Never	474 (65.2)
At times	166 (22.8)
Yes	74 (10.2)
Prefer not to answer	13 (1.8)

### Regional disparity

Among the respondents, 73.0% had a good impression of working in regional areas. The two most frequent positive impressions of regional areas were the number of surgeries they could perform (56.0%) and salaries (50.2%), whereas the most common negative impressions were the education system (38.7%) and QOL/WLB (32.2%).

### Working conditions, harassment, and dropout rates

The mean (± SD) salary in the last year of the program was 9.27 (± 4.30) million JPY, which is approximately equal to 66 thousand (± SD) USD in 2023. Among the respondents, 31.9% worked fewer than 80 h overtime per month and 10.6% worked more than 80 h per week (Table 7). Moreover, 28.0% of trainees answered that they did not have working-hour management in their hospital. Surgery or clinical care was paid for as overtime compensation in 84.5%. However, some tasks besides surgery were less likely to be paid for, including such surgery-related work as postoperative specimen dissection and operation-related tasks (37.1%), conference participation (28.7%), preparation for outpatient care and conferences, and making surgery records (22.6%).

**Table 7:** Work environment during the training program

	Number of respondents *n* (%)
Overtime work per month (h)	
≦40	27 (3.7)
41–80	205 (28.2)
81–120	288 (39.7)
121–160	97 (13.4)
> 160	77 (10.6)
Unknown	32 (4.4)
Night duty days per month [mean (SD)]	5.6 (3.2)
Working hour management	
Strictly managed (time cards, etc.)	102 (14.1)
Inadequately managed	411 (56.8)
Not managed	203 (28.0)
Do not know	8 (1.1)
Correspond to overtime allowance (multiple answers allowed)	
Surgery or clinical care	614 (84.5)
Surgery-related work (specimen preparation and operation-related tasks)	270 (37.1)
Conference participation	209 (28.7)
Preparing for outpatient care, preparing for conferences, making surgery records	164 (22.6)
Academic activities	29 (4.0)
No compensation for overtime work	58 (8.0)
Not sure which activities were considered as overtime work	61 (8.4)
Others	16 (2.2)

As many as 41.5% of the respondents reported that they had experienced some kind of harassment by the attending surgeons, and 33.0% answered that they had pondered about giving up their surgery training. The two most common reasons by far for considering quitting were poor QOL and WLB (51.1%) and harassment (50.4%).

After completing each training, 56.6% continued to work in the same area. The top three priorities for choosing the next institution were education (17.0%), geographic location (16.3%), and the number of surgeries (15.7%). Compromised QOL and WLB were documented more frequently after (9.5%) than before (1.4%) training.

## Discussion

This is the first nationwide survey to investigate various aspects of the current surgical training system in Japan. The survey investigated the backgrounds of the trainees, their motivations for embarking on surgical training, their evaluation of the training system, and the working conditions they faced during the training. The results showed that the overall satisfaction with the training system was high. More than half of the respondents (54.1%) decided before graduating from medical school to pursue surgery as their specialty and most (84.6%) expressed satisfaction with the training program. However, there were some significant issues with the training environment. Notably, 68.1% of respondents reported working more than 80 h of overtime per month [14] and 41.5% reported harassment by attending surgeons. Moreover, harassment and compromised QOL/WLB were the two leading causes for considering dropout. These findings provide valuable insights into the present status and future goals of the surgical training system in Japan.

Most of the trainees (84.6%) were satisfied with their training program. Therefore, the currently offered surgical cases appear to meet the trainees’ demands. Moreover, the survey found that the degree of satisfaction correlates with the number of surgeries each trainee performed and the fact that the surgical applicants were attracted to surgical procedures supports this finding. A previous survey in 2016 showed that self-competency was related to the number of surgeries performed [7, 8], which underscores this evidence. However, the current survey showed that when the number of surgeries surpassed 500, the overall satisfaction decreased slightly, which might be due to overwork and compromised QOL. Further analysis is needed to find the optimal number of surgical procedures for the training period.

While the satisfaction rate was high, long working hours were a fundamental problem in the training system. Values are changing now, to avoid long working hours and to prioritize the QOL of each medical doctor in Japan. As part of the workstyle reform, the Ministry of Health, Labour and Welfare of Japan has limited the working hours for medical doctors and implemented the reformed policy in April, 2024 [14]. Nevertheless, 63.6% of the respondents worked over the limit stipulated by this policy and 10.6% worked more than 80 h per week, which is the policy limit in the USA, and still used for the assessment of each program. This policy was adopted after considering the medical errors attributed to overwork [15]. The negative relationship between long working hours and academic ability, as described previously [16], might be reflected in the shortage of research time reported by 47.3% of our respondents. Another serious problem revealed by our study is an insufficient or complete lack of working-hour management. As many as 28.0% of trainees did not receive attendance or working-time management, which increases their risk of being overworked. The surgical trainees might feel obliged to cover various tasks in addition to surgical training [5]. Being exposed to or feeling obliged to comply with this conservative view of a surgeon`s working style, which contradicts the recent changes in societal values, might perpetuate the growing reluctance of medical students and junior residents to pursue an interest in the field of surgery.

Another important revelation of this survey was that 41.5% of respondents experienced some kind of harassment in each surgical training program. However, our survey was only able to get response from around half of the graduating residents, making it difficult to assess the true percentage of trainees experiencing harassment. Moreover, in this survey, instances of harassment were reported subjectively by respondents, based on their personal interpretation of the term “harassment”, similar to past research in this field [6, 17, 18]. It should be acknowledged that while this figure may include incidents unequivocally recognized as harassment under objective standards, it might encompass a range of behaviors, such as stern but educational reprimands, reflecting the diverse interpretations of ‘harassment’ in the contemporary era. The Japanese Ministry of Health, Labour, and Welfare reported that the prevalence of workplace harassment and sexual harassment is 31.4% and 10.2% in Japan [19]. The current survey revealed that harassment was one of the main reasons for trainees considering dropping out. The prevalence of harassment in surgery has been reported in various institutes and has been linked to deteriorating mental health and suicide [20]. Furthermore, harassment was described as a risk factor for medical errors and a worse patient prognosis [21, 22]. The JSS is committed to addressing harassment by fostering awareness and understanding, developing educational frameworks for mentors, prioritizing trainee wellness, and refining daily instructional methods.

Yet another issue highlighted by our survey is that while opportunities for technical skill training are improving, opportunities for non-technical skills training, off-the-job training, and didactic material training remain unsatisfactory. Non-technical skill training is described as useful, but the current survey shed light on its low attendance rate (22.9%). A non-technical skill program for surgical trainees has not been developed by the JSS. Moreover, 36.3 to 55.1% of the respondents had not used the education materials developed by the JSS. Providing online streaming and short summary videos [23] could potentially enhance usability and effectiveness.

This survey has several limitations. First, although the response rate was a robust 53.8%, we could not collect the answers of nearly half of the certified trainees. Second, it did not include trainees who had already dropped out of the training or remained uncertified. These limitations were likely to have caused a selection bias and an underestimation of the problems with the surgical training programs in Japan. Third, although we performed several simulation tests to prevent technical errors and limited the number of questions as much as possible to reduce satisficing, the actual reliability of each answer is unknown. Finally, this paper presents a descriptive analysis of the present overall status of the surgical training programs in Japan. Further subgroup analysis is needed to unveil detailed issues and gain deeper insights.

## Conclusion

This nationwide survey revealed that while the trainees were satisfied with the overall training system, significant issues such as long working hours and harassment were prevalent. Working to improve these problems could make surgery a more attractive career option for young trainees.

## Supplementary Information

Below is the link to the electronic supplementary material.

Supplementary file1 Online Resource 1 Provides a complete list of the 43 questions used in the online questionnaire survey (PDF 106 KB)

## References

[1] Japan Surgical Society [in Japanese]. https://jp.jssoc.or.jp/. Accessed 1Sept 2023.

[2] Japanese Medical Specialty Board [in Japanese]. http://jmsb.or.jp/. Accessed 1 Sept 2023.

[3] Hagelsteen K, Pedersen H, Bergenfelz A, Mathieu C. Different approaches to selection of surgical trainees in the European Union. BMC Med Educ. 2021;21:363. 34193137 10.1186/s12909-021-02779-5 PMC8243060

[4] Tomizawa Y, Miyazaki S, Matsumoto T, Uetsuka Y. Selection of and retention in surgical specialty during early career in Japan. Tohoku J Exp Med. 2020;252:95–102. 32938839 10.1620/tjem.252.95

[5] Takami H, Kodera Y, Eguchi H, Kitago M, Murotani K, Hirano S, et al. Results of an online survey of qualified teaching hospitals that take part in the surgical training programs for board certification by the Japan Surgical Society. Surg Today. 2023. 10.1007/s00595-023-02732-7. 37193795 10.1007/s00595-023-02697-7 PMC10764368

[6] Schlick CJR, Ellis RJ, Etkin CD, Greenberg CC, Greenberg JA, Turner PL, et al. Experiences of gender discrimination and sexual harassment among residents in general surgery programs across the US. JAMA Surg. 2021;156:942–52. 34319377 10.1001/jamasurg.2021.3195 PMC8319819

[7] Poudel S, Hirano S, Kurashima Y, Stefanidis D, Akiyama H, Eguchi S, et al. A snapshot of surgical resident training in Japan: results of a national-level needs assessment survey. Surg Today. 2019;49:870–6. 31102022 10.1007/s00595-019-01819-4

[8] Poudel S, Hirano S, Kurashima Y, Stefanidis D, Akiyama H, Eguchi S, et al. Are graduating residents sufficiently competent? Results of a national gap analysis survey of program directors and graduating residents in Japan. Surg Today. 2020;50:995–1001. 32125504 10.1007/s00595-020-01981-0

[9] Hashimoto D, Poudel S, Hirano S, Kurashima Y, Akiyama H, Eguchi S, et al. Is there disparity between regions and facilities in surgical resident training in Japan? Insights from a national survey. Surg Today. 2020;50:1585–93. 32488479 10.1007/s00595-020-02037-z

[10] Hida K, Hirano S, Poudel S, Kurashima Y, Stefanidis D, Hashimoto D, et al. The degree of satisfaction and level of learning in male and female surgical residents: a nationwide questionnaire survey of graduating residents in Japan. Surg Today. 2023. 10.1007/s00595-023-02683-z. 37162584 10.1007/s00595-023-02683-z

[11] Okoshi K, Nomura K, Taka F, Fukami K, Tomizawa Y, Kinoshita K, et al. Suturing the gender gap: income, marriage, and parenthood among Japanese Surgeons. Surgery. 2016;159:1249–59. 26830072 10.1016/j.surg.2015.12.020

[12] Kawase K, Nomura K, Tominaga R, Iwase H, Ogawa T, Shibasaki I, et al. Analysis of gender-based differences among surgeons in Japan: results of a survey conducted by the Japan Surgical Society. Part 1: working style. Surg Today. 2018;48:33–43. 28634729 10.1007/s00595-017-1556-0

[13] Kawase K, Nomura K, Tominaga R, Iwase H, Ogawa T, Shibasaki I, et al. Analysis of gender-based differences among surgeons in Japan: results of a survey conducted by the Japan Surgical Society. Part. 2: personal life. Surg Today. 2018;48:308–19. 28921482 10.1007/s00595-017-1586-7

[14] Ministry of Health, Labour and Welfare. Work style reform of medical doctors [in Japanese]. https://www.mhlw.go.jp/content/10800000/000516867.pdf. Accessed 1 Sept 2023.

[15] Steinbrook R. The debate over residents’ work hours. N Engl J Med. 2002;347:1296–302. 12393837 10.1056/NEJMhpr022383

[16] Barden CB, Specht MC, McCarter MD, Daly JM, Fahey TJI. Effects of limited work hours on surgical training. J Am Coll Surg. 2002;195:531–8. 12375759 10.1016/s1072-7515(02)01242-5

[17] Dyrbye LN, West CP, Sinsky CA, Trockel M, Tutty M, Satele D, et al. Physicians’ experiences with mistreatment and discrimination by patients, families, and visitors and association with burnout. JAMA Netw Open. 2022;5: e2213080. 35587344 10.1001/jamanetworkopen.2022.13080 PMC9121189

[18] Gianakos AL, Freischlag JA, Mercurio AM, Haring RS, LaPorte DM, Mulcahey MK, et al. Bullying, discrimination, harassment, sexual harassment, and the fear of retaliation during surgical residency training: a systematic review. World J Surg. 2022;46:1587–99. 35006329 10.1007/s00268-021-06432-6

[19] Ministry of Health, Labour and Welfare [in Japanese]. https://www.mhlw.go.jp/stf/newpage_18384.html Accessed 9 Dec 2023.

[20] Fahrenkopf AM, Sectish TC, Barger LK, Sharek PJ, Lewin D, Chiang VW, et al. Rates of medication errors among depressed and burnt out residents: prospective cohort study. BMJ. 2008;336:488–91. 18258931 10.1136/bmj.39469.763218.BE PMC2258399

[21] Halim UA, Riding DM. Systematic review of the prevalence, impact and mitigating strategies for bullying, undermining behaviour and harassment in the surgical workplace. Br J Surg. 2018;105:1390–7. 30007001 10.1002/bjs.10926

[22] Guo L, Ryan B, Leditschke IA, Haines KJ, Cook K, Eriksson L, et al. Impact of unacceptable behaviour between healthcare workers on clinical performance and patient outcomes: a systematic review. BMJ Qual Saf. 2022;31:679–87. 35046101 10.1136/bmjqs-2021-013955

[23] AlHasan AJMS. The power of YouTube videos for surgical journals. Surgery. 2023;174:744–6. 37419760 10.1016/j.surg.2023.05.042

