# Supplementary material for: Current status of the surgical training system in Japan: results of a nationwide questionnaire survey of graduating surgical trainees
Source: Surg Today. 2024 Jun 26;55(1):90–8. doi: 10.1007/s00595-024-02884-0 (PMC11717843; doi:10.1007/s00595-024-02884-0)
Supplement: Supplementary file 1 — Supplementary file1 Online Resource 1 Provides a complete list of the 43 questions used in the online questionnaire survey (PDF 106 KB) [file 595_2024_2884_MOESM1_ESM.pdf]

Background

1. When was your medical license issued?
( )(year)

2. What is your current age group?
a. <31 years old
b. 31-35 years old
c. 36-40 years old
d. >40 years old

3. Which year did you apply for the Board-Certified Surgeon?
a. 2021
b. 2022

4. Gender
a. Male
b. Female
c. Other
d. Prefer not to say

5. Marital status
a. Married or in a domestic partnership
b. Single or no partner
c. Prefer not to say

6. Do you have children?
a. Yes
b. No
c. Prefer not to say

7. Which of the following is your spouse's or your partner's work schedule?
a. Full-time
b. Part-time
c. Unemployed
d. Prefer not to say
e. Other _______

8. What is the size of your hometown?
a. Major city (1 million people or more)
b. Regional city (over 50,000 people)
c. Underpopulated area (under 50,000 people)
d. Other _________

9. In which area did you receive most of your surgical training?
a. Major city (1 million people or more)
b. Regional city (over 50,000 people)

c. Underpopulated area (under 50,000 people)
d. Other _________

10. Were you affiliated with a university during your training?
a. Yes
b. No

11. Please select the amount of practice time for each facility size. Please answer a to c, with i - vi.
a. University hospital
b. Tertiary emergency medical institutions other than university hospitals
c. Secondary or lower emergency medical institutions other than university hospitals

i. None
ii. <7 months
iii. 7 months - 1 year
iv. 1 year 1 month - 2 years
v. 2 years 1 month - 3 years
vi. >3 years

12. How many surgeries did you perform under general anesthesia during your training?
a. <101
b. 101-200
c. 201-300
d. 301-400
e. 401-500
f. >500

13. Which subspecialty are you primarily pursuing?
a. Gastrointestinal surgery
b. Respiratory surgery
c. Cardiovascular surgery
d. Pediatric surgery
e. Breast surgery
f. Endocrine surgery
g. Emergency surgery (Acute care surgery)
h. Not obtaining any subspecialty
i. Undecided
j. Other ______

Motivation and Timing for Choosing Surgery

14. When did you decide to become a surgeon?
a. Before entering medical school
b. After entering medical school but before clinical training
c. During clinical training in medical school

d. After clinical training in medical school
e. During the first year of initial clinical residency
f. During the second year of initial clinical residency
g. After the initial clinical residency
h. Other _______

15. How did the following factors influence your decision to become a surgeon? Please answer a to i, with A, B, or C.
a. Image of surgeons (from dramas, comics, celebrities, acquaintances, internships, etc.)
b. Surgical procedures and techniques
c. Educational system, Process until independence
d. Salary
e. Quality of Life (QOL), Work-life balance
f. Career path of a surgeon
g. Recruitment from surgeons
h. Family or relatives
i. Other _______

A) Positive influence / Reason for choosing surgery
B) Neutral
C) Negative influence / Reason for hesitating to choose surgery

Regarding the Training Program

16. What was the most influential factor in choosing the training program facility?
a. Positive atmosphere of the department
b. Number of surgeries performed
c. Variety of surgical cases
d. Comprehensive educational system
e. Income
f. Quality of Life (QOL), Work-life balance
g. Reputation of the facility
h. Recommendation from surgeons
i. Recommendation from family/relatives
j. Location/region of the training facility
k. Alma mater
l. Regional quota admission, scholarship quota, Academic loan program (Jichi Medical University, National Defense Medical college, University of Occupational and Environmental Health, etc.)
m. Other _______

17. How many papers have you written as the lead author? (Regardless of the type of manuscript)
a. 0
b. 1
c. 2

d. 3
e. 4
f. >5

18. What do you think you are lacking to write a paper?
a. Medical knowledge
b. Practical knowledge of research (how to write and submit papers)
c. Data, cases
d. Mentor
e. Time
f. Colleagues to co-write the paper
g. Motivation, Interest
h. Nothing
i. Other _______

19. How useful are the following materials for daily clinical practice and research? Please answer a to h, with 1 - 5.
a. Overview and treatment videos distributed by the Japan Surgical Society
b. Surgery DVDs provided by the Japan Surgical Society
c. Trauma workshop videos distributed by the Japan Surgical Society
d. Clinical research videos distributed by the Japan Surgical Society
e. Overview and treatment videos distributed by organizations other than the Japan Surgical Society (other societies, companies, publishers, SNS etc)
f. Surgery videos distributed by organizations other than the Japan Surgical Society
g. Japanese Advanced Trauma Evaluation and Care (JATEC) Course
h. Clinical research videos distributed by organizations other than the Japan Surgical Society

1. Very useful
2. Useful
3. Not useful
4. Not useful at all
5. I have not taken it, so I cannot evaluate

20. Please evaluate the <u>clinical skills (surgery, diagnosis)</u> of the main attending surgeons you met during training.
a. Extremely high
b. High
c. Not very high
d. Low

21. Please evaluate the <u>education skills (surgery, diagnosis)</u> of the main attending surgeons you met during training.
a. Extremely high
b. High
c. Not very high
d. Low

22. During your training, did you receive training in surgical techniques outside the operating room? (Dry lab, Wet lab, Animal lab, Cadaver, etc.)
a. Received regularly
b. Received irregularly
c. Had a chance, but did not
d. Did not have any chance
e. Don't know/Not sure

23. During your training, did you receive training in non-technical surgical skills? (Coaching, Team STEPPS, Team training, etc.)
a. Received regularly
b. Received irregularly
c. Had a chance, but did not
d. Did not have any chance
e. Don't know/Not sure

24. What do you think are the problems with the Japan Surgical Society's Surgical Training Program? (Multiple answers allowed)
a. The content of the program is not understood by trainees
b. The experience in daily surgical clinical practice does not match the program's goals.
c. Not receiving appropriate feedback from attending surgeons
d. Achievement of goals are not evaluated
e. The training satisfaction of the facility is not evaluated
f. I don't think there are any particular problems
g. Other _______

25. In the minimum limit number of experience cases designated for each area of the Surgical Training Program, which area was most difficult to experience?
a. Gastrointestinal and Abdominal Organs (50 cases)
b. Breast (10 cases)
c. Respiratory System (10 cases)
d. Heart and Great Vessels (10 cases)
e. Peripheral Vessels (10 cases)
f. Head and Neck, Dermatology, Endocrine Surgery (10 cases)
g. Pediatric Surgery (10 cases)
h. Trauma (10 points)
i. Endoscopic Surgery (10 cases)
j. I was able to experience all areas without any difficulty
k. I don't know

26. Have you ever been harassed by the attending surgeons? (Power harassment, Moral harassment, Sexual harassment, Academic harassment, Alcohol harassment, etc.)
a. Yes
b. No
c. Prefer not to say

27. Where do you work after completing your surgical training?
a. Same as the training facility
b. I have left the area/facility of training for now, but planning to return soon
c. I have left the area/facility of training and would like to return someday
d. I have left the area/facility of training and have no plans to return
e. Other _______

28. What was the highest priority when deciding your initial post-training facility or affiliation (including university medical departments)?
a. Number of surgeries performed
b. Variety of surgical cases
c. Comprehensive educational system
d. Income
e. QOL, Work-life balance
f. Location/region of the facility
g. Family circumstances
h. Alma mater
i. Regional quota admission, scholarship quota, Academic loan program (Jichi Medical University, National Defense Medical Colleges, University of Occupational and Environmental Health, etc.)
j. Currently not affiliated or working
k. Other _______

29. Are you satisfied with the surgical training you received?
a. Very satisfied
b. Somewhat satisfied
c. Somewhat dissatisfied
d. Very dissatisfied

Regional Disparity

30. What are your impressions of providing surgical care in regional hospitals?
a. I have a good impression.
b. I don't have a good impression.

31. Please select one positive impression you have of surgical treatment at regional hospitals.
a. Number of surgeries performed
b. Variety of surgical cases
c. Comprehensive educational system
d. Impact on achievements and career
e. Interpersonal relationships (Patients, Colleagues, Family, Residents, etc.)
f. Workplace environment excluding relationships (Location, Hospital size, Facilities)
g. Income
h. QOL, Work-life balance

i. Nothing
j. Other _______

32. Please select one negative impression you have of surgical treatment at regional hospitals.
a. Number of surgeries performed
b. Variety of surgical cases
c. Comprehensive educational system
d. Impact on achievements and career
e. Interpersonal relationships (Patients, Colleagues, Family, Residents, etc.)
f. Workplace environment excluding relationships (Location, Hospital size, Facilities)
g. Income
h. QOL, Work-life balance
i. Nothing
j. Other _______

33. What information or conditions would make you consider working as a surgeon in the countryside? - Free response (______)

Working Environment of Surgeons

34. What was the average number of overnight shifts per month during your training? (Including external assignments and part-time work.)
( ) shifts

35. During your training, what was the average monthly amount of overtime work? (Exceeding 40 hours a week, Including on-calls and external assignments) a. <41 hours (within 10 hours per week)
b. 41-80 hours (10-20 hours per week)
c. 81-120 hours (20-30 hours per week)
d. 121-160 hours (30-40 hours per week)
e. >160 hours (over 40 hours per week)

36. Was your working time managed during your training?
a. Strictly managed (using time cards or similar methods)
b. Generally managed
c. Not managed
d. I don't know

37. What was your annual income from the primary facility in the final year of your training?

( ) x 1 million yen

38. In which of the following tasks were you payed for overtime payment during your training? (Multiple choices allowed)
a. Clinical work and surgery

b. Tasks related to surgery (Specimen preparation, ork outside operations)
c. Participation in conferences
d. Tasks related to medical records (Preparation for outpatient visits, Conference preparation, Surgical records, etc)
e. Academic activities (Presenting at conferences, creating slides, Writing papers, etc)
f. There was no paying for overtime work
g. I am unsure about which activities were considered as overtime work
h. Other: ________

39. Have you ever considered quitting the training program?
a. I have never thought about it.
b. I have thought about it a little.
c. I have seriously thought about it.
d. I prefer not to answer.

40. (Please answer only if you chose b or c in the previous question.)
Why did you consider quitting?
a. The actual experience was different from what I imagined.
b. Couldn't master surgical techniques, Unspecifiedcomplications occurred.
c. I was not able to receive sufficient education as a surgeon.
d. Income was different from what I respected.
e. Low quality of life, Poor work-life balance.
f. I felt uncertain about my career.
g. Poor relationships with others (Bullying, Harassments, etc.).
h. I found a more attractive medical specialty or a job.
i. Family circumstances.
j. Health problems (Physical, Mental health).
k. Other: ________

41. (Please answer only if you chose b or c in the previous question.)
What specific career changes did you consider?
Free response: (   )

Other

42. If you have any opinions regarding your training or training program, please feel free to write them down.
Free response: (   )

43. Lastly, if you have any opinions about the survey, please feel free to write them down.

Free response: (   )
